# Why is now the best time for approaching the significant relation between “Life and Medicine”

**Published:** 2008-02-25

**Authors:** Purcărea Theodor

**Affiliations:** * Romanian – American University President of the Romanian Distribution Committee Member of the Board of International Association for Distributive Trade (A.I.D.A. Brussels)

## Abstract

The present paper is aimed to analyze the need for an adequate engagement in a better understanding of the relation between “Life and Medicine”, within the increasing importance of the patient's point of view, while quality of life is managed within medicine and even going beyond. It is necessary to reconsider the essential role of communication in an adequate offering of the right answers in accordance with the changes in medical care, quality of life being one benefit. Science should inform the news agenda on the way of building a collaborative environment, turning up the conversation focused on patients' lives, while practicing medicine with an open heart for the physician-patient-family relationship and based on models of quality of life outcomes. That is why it is important to create new knowledge and competencies starting from intangible resources such as an engagement as a consequence of a communication programme which produces an increased level of perception of the outcomes of the necessary mature actions as meeting and surpassing patient expectations, not forgetting the vulnerability to the way in which choices are described. From here is resulting the imperative of the development of better abilities in innovation, differentiation, branding and patient-driven health care service, largely opening the window of opportunity for the educational mission to educate marketers who make a difference by taking the lead from a strategic planning position.

## 1. Living in an international medical world within the rise of modern medicine

We are already living in an international medical world, many medical business transactions being outsourced to communication centers located in different continents. Medical tourism (a new term but not a new idea for the medical services industry which is evolving quickly: “on the rise for everything from cardiac care to plastic surgery to hip and knee replacements” [**1**] is increasing [**2**] and consumers go online [**3**] to learn more about health care and drug treatments, using general health-focused Web sites (50%, in USA), going to specific ailment-focused sites (35%, in USA) and heading to branded, pharmaceutical company drug sites (13%, in USA). They are seeking information about symptoms for a specific ailment or condition (55%, in USA), are looking for tips for managing ailments and health conditions (27%, in USA) and 18% (in USA) want information on specific drugs to treat their aliments. Now already “The Doctor Is Online: Moving Toward Physician-Patient e-Visits” [**4**] and “Patients Eager To E-Mail Their Doctors” [**5**].

Medicine (up to now centered on the retrospective analysis of symptoms to uncover underlying causes) may be undergoing a profound transformation as it moves from symptom-based to cause-based diagnosis [**6**]. In April 2005, while approaching “The Holy Trinity of Health Reform” (“Costs: We spend too much and this because we can do more; Access: Insure the uninsured; Quality: Quality is uneven”), David M. Cutler [**7**] from the Harvard University argued that *there are changes in medical care and quality of life is one benefit*. Because people value their health highly, medical care would take a large share of spending increases and there are some *recommendable steps in a health reform*: cover everyone (we must never forget anyone and taking into account - as Cutler underlines- that “waste and value are the Dr. Jekyll and Mr. Hyde of health care”); transition to a pay-for-performance system, using measures of quality (process, outcomes, satisfaction) and considering lower patient cost sharing for effective care. Cutler’s vision of the future health system encompasses: less wasteful care and more valuable care, higher or lower overall spending, sustained cost increases for new technologies, ensuring a stable financing system, insuring everyone, challenges and rewards for high value.

In 2006, the chief Editor of Test & Measurement World quoted Richard Sykes - rector of the Imperial College of London and the former chairman of GlaxoSmithKline - who said: “Science should inform the news agenda, not the other way around. Before we can engage the public in an informed debate we need the scientists to do the science” [**8**]. In 2006 too, Barbara A. White [**9**] argued that in order to facilitate the goal of building a collaborative environment we need to understand and practice the skills tied to the art of diplomacy. In November 2007, Graham Hill [**10**] underlined the answer received from Chris Bucholtz pointing that: the first step in engaging someone is to show respect and courtesy; the form of marketing would actually work is toning down the selling and turning up the conversation.

## 2. The problem of quality of life in medicine

According to the new book of Professor Regina Herzlinger [11] - Who Killed Health Care? - presently medicine is more art than science and we need a change to make health care more responsive to customers, considering the human problem, the economic problem, the lack of quality and the lack of assets.

At the very beginning of this year someone posted that: “Everybody should be looking out for their own well-being to insure that they can live a long healthy life” [**12**]. While at the end of November 2007, on the occasion of Heidelberger Innovationsforum, [**13**] Dr. Klaus Heumann from Biomax Informatics AG, a german company, underlined that the value of information comes through analysis and integration with the current knowledge and quoted WellPoint’s Leonard Schaeffer: …, “when you go to the doctor, you get evidence-based medicine only 55% of the time. That means you are not getting generally accepted best practices 45% of the time …”

Within the increasing importance of the patient's point of view, *“the physicians' job description will be changed to focus on patients' lives rather than patients' bodies”*, underlined in April 2003 a representative of University of Washington [**14**], who found that “perceived health, health-related quality of life, and health-state utilities bring health assessment progressively closer to the patient's perspective,” some of the most important patient outcomes being now valid because they are subjective. 

Approaching the problem of quality of life in medicine within the lacking of a clear conceptual basis for quality-of-life measures, two representatives of the “Institut National de la Santé et de la Recherche Médicale (INSERM), Unite 292, Hôpital de Bicêtre, Le Kremlin-Bicêtre, France” [**15**] argued that: << quality of life as an outcome could be explored more clearly (ie, defined) if quality of life were replaced with a more easily handled notion such as that of *"subjective health status."* However, the idea that the patient's perspective is as valid as that of the clinician when it comes to evaluating outcomes has a great deal of legitimacy and should certainly not be abandoned>>. 

Beyond the frontier, a representative of the Department for Medical Psichology of Hamburg University, Germany [**16**] underlined that measuring instruments of quality of life are now available being increasingly applied, and “can make a contribution towards improving the care afforded the patient”.

It is a real challenge to think about the relation between Life and Medicine, when: “Patients will insist on getting sick. Time waits for no man.” [**17**] Ron Elisha, who graduated in medicine in 1975, and has spent most of his career in full-time general practice said that “medicine, like lava, has consumed everything in its path - time, energy, intellect, memory, even the very compassion from which the desire to practise it first sprang. *Nothing can escape the pervasive imperialism of the medical experience.”*

Dr. Jeffrey Geller, professor of psychiatry and director of public-sector psychiatry at the University of Massachusetts Medical School in Worcester, speaking about the importance of “practicing medicine with an open heart”, [**18**] attracted our attention underlining that men and medicine have much to learn from Jay Neugeboren who stated: “The things *people want from their doctors*, and that they are, in recent years, getting less of, have much in common with *what they want from friendship."* Geller sustain that it isn’t too late for American medicine and, point with an African proverb quoted by Neugeborens: “The best time to plant a tree is 20 years ago. The best time is now”. *Taking this into account why couldn’t we say that the best time for approaching this significant relation between Life and Medicine is now?*

According to the opinion of Nancy Kutner [**19**] (expressed in a paper presented at the annual meeting of the American Sociological Association in San Francisco, August 14, 2004), quality of life (now prominent in clinical studies) has been assailed in the medical literature as a “problem” on both conceptual and measurement grounds and its sloganization and medicalization - trends anticipated by Sol Levine (who heralded in 1987 “the emergence of quality of life as a major concern in health as a salutary development for medical sociology”) - are strategies by which the problem of quality of life is managed within medicine and, to go beyond this Kutner recommends to *“develop and test conceptual models of quality of life outcomes, a natural focus for medical sociology”*.

Two years ago, in 2006, in an interesting Book Review, [**20**] Ornella Moscucci showed her admiration for Barbara Bridgman Perkins (author of “The medical delivery business: health reform, childbirth, and the economic order”, New Brunswick, and London: Rutgers University Press, 2004), who produced “an intelligent, thought-provoking and insightful account of the business approach in medicine”, starting from revisiting the specific debate in the medical delivery business and pointing that *“business principles do not enhance efficiency without impinging on the clinical content and practice of medicine:* the practice, the science and the business of medicine are all inextricably bound up together.”

If we value our health highly is our duty to act to add life to years. If we take into account, for example, Confucian ideas (“Confucians emphasize cultivating the virtue of doctors themselves is more important than norms or ethical standards”), [**21**] we have to consider the human being as a part of a family and community, medical decision-making being made or agreed by the family as a whole, where the preferential choice of the family is what is count at the end, the informed consent being given by the family. It is very important all what is happening at the level of the physician-patient-family relationship, including from the point of view of the undergraduate being at the very beginning of its start on the “medicine way”. 

I have noted, for example, that on first January 2008 “Adam” [**22**] posted “a list of things that got him through first year of medicine: Friends; Food; Crushes; Sense of Humour; Heroes; Real Life Drama.” I think we need passion coming from purpose. But are we travelling in the right direction?

## 3. Get out in the field and understand the relation between Life and Medicine 

There is a real need for an adequate engagement in a better understanding of the relation between Life and Medicine, looking out for our own well-being within the increasing importance of the patient's point of view, while quality of life is managed within medicine and even going beyond. We all agree that are well-known common aims and we like the conversation on the necessary mature actions for, putting people first and trying to maximize our actions' potential for success, by transferring knowledge in the matter regarding what works effectively and what did not work properly. This reality thus involves a larger commitment in accepting the challenge of creative thinking, progressing through knowledge and understanding towards reciprocal trust. Education is the most important instrument for adaptation to change as an opportunity, *creating new knowledge and competencies starting from intangible resources*. Such an intangible resource is, for instance, *an engagement as a consequence of a communication programme which produces an increased level of perception of the outcomes* of the underlined necessary mature actions as meeting and surpassing patient expectations.

Eric Warner of the Russell Sage Foundation - quoted by deputy editor Craig Lambert in an editorial published by the prestigious “Harvard Magazine” [**23**] – drew attention to the fact that in behavioural economic science the choice depends on the way in which the decision maker describes the objects, there being a vulnerability to the way in which choices are described. In Lambert’s opinion: the eclipse of the hyper-rational Economic Man opens the way for a model of a wealthier and more realistic human being on the market, where the brain, with all its classic instincts and vulnerabilities can be both a predator as well as prey; the models of behavioural economic science can help in the design of a company with more compassion for the creatures whose strong and weak points evolve under much simpler conditions.

There is a market for everything (for example, to gain experience medical students use cadavers) [**24**]. Professor Regina Herzlinger - the mentioned author of the book “Who Killed Health Care?” - popularized the term “consumer-driven health care”, a common way to describe a new health care solution, by converting the entire health care system to one that is responsive to the patient as the ultimate consumers of its goods and services (“empowering individuals and bringing their force to bear on the offerings of doctors, hospitals, and insurance and pharmaceutical companies”) [**25**]. Richard Hamermesh, faculty chair of Harvard Business School's Healthcare Initiative raised the question of this “underperforming industry”, arguing that: “…the name of the game is supposed to be quality up and cost down… it's been very slow with the quality movement”. Within this framework, Sean Silverthorne quotes the former CEO of Merck, Gilmartin, who said that: twenty years ago few policymakers believed that markets could play a role in improving health care; the major players seem increasingly receptive to rethinking the system; health care is an opportunity for the educational mission to educate leaders who make a difference in the world [**26**].

In may 2005, being in Bucharest, the “father of marketing”, Philip Kotler, attracted our attention to the imperative of “the development of better abilities in innovation, differentiation, branding and service, in a word Marketing”. In Kotler’s opinion marketing is the art of brand building, what is not is a brand is goods and then the price is everything, the only winner being the low-cost producer. New technologies place marketing in the situation of becoming invisible, the result being a closed-loop business where an increase or a decrease of marketing expenses can be directly connected to income and profit. In fact, the capacity for strategic innovation and imagination are coming from the assembly of instruments, processes, abilities and measures which will allow the company to generate more and better ideas than their competition. This on the base of assuming social responsibility because the success of the business and the continuous satisfaction of the client (the patient) and other affected parties are closely connected to the adoption and implementation of high leadership standards of a business and marketing.

*“Marketing takes the lead”*, by *“walking in the patient’s shoes”* and by *“seeing through the patient’s eyes”*, tells us the president of a firm specialized in strategic Web-based communications for small to medium-size health care providers (E-savvy Communications, Jefferson City, MO., USA). Barbara S. Long [**27**] approached the case of the so called *“experience mapping”*, which is different from process mapping and typical patient satisfaction research: hospitals are using the knowledge to tweak services and facilities to improve satisfaction and drive more business; a strategic shift in focus from operations to customer; hospitals are starting to see the value in truly understanding the patient experience; one of the first to try experience mapping was the University of Texas M.D. Anderson Cancer Center in Houston; experience mapping helps patients recall their specific interactions with the organization; sometimes the research identifies challenges the hospital did not know existed; reviewing the results with staff and developing a plan to operate changes constitutes the final step in an experience-mapping project, the ultimate reality check being to weigh the benefit and the costs to create the “ideal” patient experience etc. *“Experience mapping”* explains the emotions behind the actions and *helps developing a strong customer-centric focus, marketing departments being the ones taking the lead from a strategic planning position.*

Paraphrasing Kotler’s mentor, Peter Drucker (the “father of management”), we can say that the purpose of health care services marketing is to know and understand the patient so well that the product or service is suited to him and sells itself. Patients respond differently to the image of a company and a brand, the identity (the way in which a company identifies itself, self-positions itself or positions its products) and the image (the way in which the public perceives the company or its products/services) requiring their distinction. The key to branding, emphasized Kotler (who reminded in Bucharest of the “Y&R” model of brand strength: vitality and stature) is the perception by customers of the differences between the brands of a category of products. Present in Bucharest, in May 1998, with the occasion of the International AIDA Brussels Congress (organised by the Romanian Distribution Committee – CRD, affiliated to AIDA), William S. Webb, the Director of the London Institute of Distribution Management, again drew attention to the fact that: “the legitimacy of the brand depends on consistency and continuity, otherwise clients cease to believe you.” And within “patient-driven health care” we have to take huge care because as underlined by Susan Solomon - author of the book “Building powerful health care brands” - “health care branding is an entirely different animal” [**28**]. 
